# A conceptual framework for the ethical analysis of moral conflicts in migrant live-in care

**DOI:** 10.3389/fpsyt.2024.1453948

**Published:** 2025-01-10

**Authors:** Milena von Kutzleben, Merle Weßel, Natalie Ulitsa, Anna-Eva Nebowsky, Liat Ayalon, Mark Schweda

**Affiliations:** ^1^ Division for Prevention and Rehabilitation Research, Department for Health Services Research, School VI - Medicine and Health Sciences, Carl von Ossietzky Universität Oldenburg, Oldenburg, Germany; ^2^ Diversity Executive Department, Hannover, Germany; ^3^ Louis and Gabi Weisfeld School of Social Work, Bar Ilan University, Ramat Gan, Israel; ^4^ Division for Ethics in Medicine, Department for Health Services Research, School VI - Medicine and Health Sciences, Carl von Ossietzky Universität Oldenburg, Oldenburg, Germany

**Keywords:** moral conflicts, ethical analysis, care ethics, norms & moral standards, values, family caregiving, live-in care, dementia

## Abstract

In many industrialized countries, hiring a migrant live-in carer represents a promising solution to support families caring for an older person at home and to avoid institutionalization. Migrant live-in carers live in the household of the person in need of care and provide extensive care and social support. They usually come from geographic areas such as Eastern Europe or Southeast Asia. Due to often unclear legal regulations regarding labor and migration status, as well as contradicting expectations and entangled vulnerabilities within the triad of the person in need of care, the live-in carer, and the family, these live-in care arrangements are prone to a variety of moral conflicts that require ethical analysis. This article proposes a conceptual ethical framework for analyzing moral conflicts within live-in care arrangements. By recognizing and addressing these conflicts within the multi-level ethical framework, the ground for a triadic perspective is laid and the ethical discussion around live-in care for older people can be put on an empirical basis. This can help to inform counselling and support for these arrangements, as well as policy advice for ethical solutions and improved caregiving practices.

## 1 Introduction

Aging in place is a leading paradigm for later life around the globe (1) and most older people live at home. Usually, care responsibilities lie in the hands of relatives like spouses or children (2). Even if public support is offered, for example, by long-term care insurance schemes, everyday care and support needs may not be covered sufficiently. For example, families affected by dementia are confronted with extensive care requirements – often around the clock. In this situation, hiring a migrant live-in carer can appear as a promising solution to support families and to avoid institutionalization. These live-in carers usually come from abroad, for example, Eastern Europe or Southeast Asia, and live with the persons in need of care in their home. They provide extensive social and often also nursing care and are usually expected to be available 24 hours a day (3).

Migrant live-in care is a widespread model of care in many countries, but the arrangements are often shaped by structural disparities. The empirical body of evidence points to a considerable potential for problems related to the precarious social and legal situation of the live-in carers (4, 5). To a lesser extent, the perspectives of relatives (6–8) or of older persons with and without dementia have been taken into account (9–11). However, research to date has provided little insight into the complex triadic constellation of live-in carer, relatives, and care recipient. A triadic perspective is important to understand the complexity of moral issues in live-in care. Furthermore, the specific structure of moral conflicts that arise in this context deserve closer ethical examination (12, 13). Live-in care arrangements can be characterized by entangled vulnerabilities and often contradicting needs, wishes and expectations of the parties involved (14). For example, conflicts can be rooted in colliding with personal interests or moral orientations, in disagreements regarding the allocation of care responsibilities and matters of workplace organization, or different understandings of the care needs and good care of the person in need of care (4). They may be open conflicts that are explicitly discussed or implicit conflicts that are never expressed, but usually affect the quality of care as well as the satisfaction and well-being of people in need of care, family carers as well as live-in carers.

In this paper, we introduce a conceptual framework to identify and analyze moral conflicts in live-in care arrangements from an ethical perspective. In doing so, we follow an approach of empirically informed ethics that pursues a systematic combination of ethical analysis and social research (15). The proposed framework aims to facilitate a first ethical categorization of moral conflicts in this area and is therefore neither committed to a specific ethical theory, such as utilitarian ethics, deontological ethics, or care ethics, nor to specific cultural contexts or national framework conditions. We distinguish collisions between moral norms and values which can constitute different types of conflict occurring on different levels (see **Table 1**). In this way, we provide a heuristic tool for the empirical analysis and ethical evaluation of these conflicts. Using examples found in own empirical research and described in the work of colleagues, we illustrate how this tool to develop a more profound and differentiated understanding of concrete moral conflicts that occur in the context of live-in care.

**Table 1:** Types of moral conflicts in migrant live-in care arrangements on different levels.

	Types of moral conflicts		
	Norms vs. norms	Values vs. values	Norms vs. values
1) Micro level			
*Intra-individual*	Live-in carer’s respect for the care-dependent person’s autonomy vs. protection and safety (e.g. when the person has dementia)	Working as a live-in carer as a trade-off decision: improving living standard of one’s own family but leaving family members behind in the home country	Adequate fulfillment of the care-dependent person’s needs (around the clock care) collides with live-in carer’s (workers) rights
*Inter-individual*	Collision of cultural norms of care and understanding of ageing between the relatives and the live-in carer	Religious beliefs of the live-in carer collide with that of the person in need of care or family caregiver’s values and life style (e.g. concerning sexuality)	Relatives’ or care dependent person’s expectations regarding caregiving behavior of the live-in carer (e.g. norms of care responsibility) may clash with the live- carer’s interest in personal wellbeing (e.g. privacy, enjoying free time)
2) Meso level			
*Individual vs. family/agency*	The perceived role and expectations of family members regarding live-in care may be in conflict with the perceptions and expectations of the live-in carers or the agency	Values promoted by an agency (e.g., exchangeability of carers) may conflict with values of live-in carer (e.g. emotional bond with person in need of care or family)	Family values of unconditional care are at odds with the live-in carer’s right to individual autonomy and privacy
3) Macro level			
*Individual vs. state policy/law*	National labor law regulations may collide with contractual agreements and familial expectations	State laws manifest values (e.g. regarding the weighing of individual autonomy and privacy) that are at odds with live-in carer’s values (e.g. stronger emphasis on care and family relations)	Benefits from long-term care insurance are not sufficient to cover for a fair live-in care arrangement, which leads to a violation of family values and feelings of guilt

## 2 Background

Live-in care is a common form of care in most Western industrialized countries, but also countries like Taiwan or Singapore rely on this model. All these countries have an aging population, but limited means to provide adequate long-term care structures and services. Yet, they differ regarding migration processes, employment regulations, and long-term care regimes (16). While some countries such as Israel (live-in carers mainly come from the Philippines as well as from India, Sri Lanka, Moldova or Uzebkistan) and the UK (live-in carers mainly from the Gulf States, but also from India, the Philippines and Indonesia) strictly regulate the length of stay in the country, Canada (live-in carers mainly from the Philippines), offers a path for legal migration via the *Live-in Carergiver Program* and opens up the possibility to gain citizenship (17, 18). In Germany and Switzerland, live-in carers mainly come from Eastern European or Baltic countries and usually live in shuffle migration, travelling back and forth between the live-in arrangement and their home (18, 19). Both countries are lacking standardized pathways and clear legal frameworks. The regulations are hardly transparent for families and live-in carers and often live-in care takes place in a legal “grey area” or is actually illegal under labor or criminal law (20). In Austria (live-in carers mainly from Slovakia and other Eastern European countries), live-in care is legally covered under the *Constitutional Law on Care* and within the free movement directive of the European Union. However, regardless of specific national regulations, the live-in care model leads to problematic arrangements in terms of working hours and conditions (18).

In many countries, like Switzerland, Germany or the Netherlands, placement agencies play a crucial role and have a significant influence on the dynamics in live-in care arrangements (18, 21, 22). They are hiring live-in carers and are supposed to offer support for relatives to navigate legal questions and find the right live-in carer for the care recipient considering specific needs and support the live-in carer with the migration process. In some cases, they also provide training in care skills and knowledge and function as moderators in the case of conflicts or abuse (22, 23). However, in practice families report that communication and agreements with agencies are often unreliable and cause conflicts, insecurity and crises. For example, live-in carers do not always receive the information that the cared-for person has dementia, or families are falsely informed that the live-in carer is experienced in dementia care and speaks the local language. Especially in systems like Germany, where the live-in carers change on a regular basis, relatives as well as live-in carers face the challenge of coming to terms with each other and with the person in need of care, and both parties often feel betrayed. They feel left alone by “their” agency as they do not receive the expected or even promised supervision and guidance (8, 14, 24). Especially if the cared for person has dementia, the constant change of live-in cares and the associated uncertainties and discontinuities can be experienced as a permanent crisis (24).

Live-in care arrangements are often burdened by severe structural disparities and problems, regardless of the country or the mediation through a placement agency. Gender-sensitive migration research points out to the fact that the vast majority of live-in carers is female. They usually come from economically poorer countries and the live-in care migration provides an opportunity to support themselves and their families at home. The migrant live-in care model supports care chains and care drains where the care systems of the countries of origin are drained from their informal care resources (16, 25). Especially in countries where live-in care is part of the grey care market, the doors are open to exploitation, for example, when no regular social security is provided or when the contracts of the live-in carers include provisions and contractual penalties (26). Families and live-in carers are often left alone to negotiate working conditions, and both have few legal options when problems with payments or working hours occur (5, 8). Live-in carers report long working hours, sometimes no free time for weeks or months, and physical abuse from the person with dementia (21, 23). The employment relationship is entangled with a family relationship between the relatives, the live-in carer and the person in need of care, especially if the care arrangement lasts for a long time and the persons involved develop a close emotional relationship (27, 28). In consequence, professional and personal lines are blurred and families’ expectations towards the live-in carer are often diffused or ambivalent (14). Although activities are usually defined by a contract, this is overlaid by “collateral contractual mechanisms” fostered by the informality of the domestic setting (8). All this can lead to issues of responsibilities, guilt, and power structures (21, 23, 27).

## 3 The development of a conceptual framework for ethical analysis of moral conflicts

While existing social research points to a high potential for, and broad variety of, serious grievances emerging in the context of live-in care, it usually does not provide any explicit theoretical account of their moral offensiveness and objectionability. What is missing is a perspective that can explain in a differentiated way what exactly is problematic about the respective phenomena and thus allows to specify and justify their critique or condemnation (12). In this contribution, we start from the assumption that many of these issues ultimately point to underlying moral conflicts in live-in care that call for a closer ethical analysis.

From a philosophical point of view, a moral conflict describes a situation in which moral principles, obligations, and/or duties collide. The question to what extent decision-making and work situations of carers can cause moral issues has received considerable attention in nursing studies (29–35). However, the pertinent contributions usually subsume the respective issues under “moral distress” and leave their concrete moral structure and scope unexamined. Furthermore, the focus is often on professional nurses and care workers in a formal care setting and less on informal carers and informal domestic care settings. Expanding the focus beyond this domain is a necessary step to examine moral conflicts within live-in care arrangements.

In order to prepare the ground for a systematic ethical characterization and categorization of the different kinds of moral conflicts that can arise in a live-in care arrangement, we first need to distinguish different understandings of morality. In a general, descriptive sense, “morality” refers to judgments or standards regarding what intentions, actions, or institutional structures and processes are to be considered as good, right, or proper (36, 37). For a long time, moral philosophy and especially applied ethics was based on a rather narrow understanding of morality in terms of strict moral claims and obligations that individuals or groups have towards themselves or vis-a-vis each other, e.g., the claim to be treated with respect or the duty not to hurt others. This perspective can be called normative since it refers to moral norms, that is, general rules or standards of moral acceptability like the rule to respect others and avoid harm (37).

However, in more recent years, this narrow normative focus has been criticized since it neglects important moral questions regarding individual happiness, fulfilment and flourishing, e.g., what is desirable and important in life and gives our existence value and meaning (38, 39). This perspective can be called evaluative as it is not so much concerned with prescriptive norms of what is morally right or wrong but with eudemonistic standards of what is good, valuable and meaningful in life. It is important to note that both perspectives are not mutually exclusive but frequently closely entwined or even interdependent: On the one hand, values regarding a good life can be implemented through a set of general norms, for example, a catalogue of human rights recognizing basic needs and protecting individual wellbeing and self-fulfillment. On the other hand, individuals and communities can value moral norms such as justice to a degree that they become a personal or collective value for them.

In order not to exclude potentially relevant dimensions and kinds of moral conflicts right from the start, it appears generally advisable to start from a broad understanding of morality that comprises normative as well as evaluative aspects and factors: moral norms as well as values (39, 40). Especially in the field of migrant live-in care, where individuals from different cultural backgrounds share the deeply personal space of daily living and are entangled in value-laden practices and relationships of care, it seems plausible to assume that moral conflicts not only involve general rights or responsibilities, but also individual ideals and cultural orientations regarding wellbeing, home and family, as well as good care (24; Zriker et al., 2024; 41–43).

Furthermore, it is important to acknowledge that moral conflicts can arise on several societal levels. In the proposed framework we differentiate between three levels (see **Table 1**): The micro- the meso- and the macro-level. On the *micro-level*, individuals may struggle with reconciling different moral norms or values. This can be *intra-individual*, but also *inter-individual* if one person’s moral norms and/or values clash with the moral norms and/or values of another person. At a *meso-level*, norms or values of institutions or organizations such as the family, the agency or a nursing service come into play and can cause conflicts in the live-in care arrangement. On a societal *macro-level*, moral conflicts in the live-in care arrangements can also involve norms and values which are connected to state policy and laws. In **Table 1**, we explain our conceptual framework along the two mentioned axes (1) Norm vs. norms; values vs. values; norms vs values and (2) Micro-, meso-, and macro-level and provide examples of possible moral conflicts (interlay between norms and values) on each level.

In the following, we illustrate paradigmatically how the proposed framework can help to develop a more profound and differentiated understanding of concrete moral conflicts that occur in the context of live-in care. We apply the framework’s ethical perspectives to exemplary situations found in existing empirical research in order to analyze and interpret them with regard to moral norms and values involved on different levels and how their contradiction leads to moral conflicts.

### 3.1 Potential moral conflicts of live-in care on the micro level

#### 3.1.1 Intra-individual conflict of norms

Many of the problematic issues of live-in care addressed in the literature apparently pertain to the micro-level of moral conflicts between members of the live-in triad. Thus, regularly reported cultural tensions between the live-in carer and the relatives can be interpreted in terms of moral conflicts between more individualistic and more paternalistic or collectivistic moral orientations. For example, family caregivers often expect live-in carers to respect their relative’s autonomy while the live-in carer may tend to restrict personal autonomy and freedom for the sake of physical safety, or the overall wellbeing of the family. In live-in care arrangements for a person with dementia this could mean hindering the person from leaving the apartment or forcing her to eat and drink (6, 14). With regard to the live-in carer’s inner conflict of norms concerning either autonomy or safety of the person they feel responsible for, this would be an intra-individual norm-conflict.

#### 3.1.2 Intra-individual conflict of values

Conflicts of values on the micro-level can be identified when live-in carers have their own distinct values that are challenging to fulfill simultaneously. For instance, the decision to work as a live-in carer in a foreign country entails a complex trade-off. On the one hand, there is a desire to pursue a better life and provide a higher standard of living for oneself and one’s family. On the other hand, it requires “sacrifice” – leaving one’s family members behind, living far away, and missing the opportunity to raise one’s own children or caring for one’s own older family members (intra-individual value-conflict) (cf. Bruquetas-Callejo, 2019).

#### 3.1.3 Intra-individual conflict of norms *vs*. values

Another type of moral conflict that can be retrieved from literature is a norm-value-conflict, which can arise when a person’s values clash with recognized moral norms. For instance, someone may hire a live-in carer to fulfill the wish of a close relative to continue living at home. At the same time this can conflict with one’s personal understanding – and acceptance of general norms – of fair working conditions. This situation is problematic as the relative or family exploits the live-in carers, neglecting their rights and disregarding established work laws as societal norms. Fulfilling the person’s needs in this context perpetuates structural inequality (7).

#### 3.1.4 Inter-individual conflict of norms

An inter-individual norm-conflict arises if the live-in carer and the relatives adhere to different (cultural) norms. For example, as described above for intra-individual conflict of norms, this can be regarding the weight given to individual autonomy of the person in need of care (8, 22).

#### 3.1.5 Inter-individual conflict of values

Similarly, conflicts of values can occur between live-in carers, family caregivers, and the person in need of care regarding for example religious values clashing with sexual values. For example, a catholic belief of the live-in carer may collide with the person’s with dementia, or the family caregiver’s values, e.g., concerning sexuality when one of them defines themselves as gay or queer. Another case with a high potential for conflicts is described when migrant live-in caregivers are employed in faith-based societies like an ultra-orthodox Jewish family in Israel (44).

#### 3.1.6 Inter-individual conflict of norms *vs*. values

An alternate combination of conflicts on the micro level that we have found in one of our own previous qualitative studies, is when a live-in carer may find that certain values held by family members violate moral norms, such as widely recognized and performed standards of due care and responsibility (14).

### 3.2 Potential moral conflicts of live-in care on the meso level

#### 3.2.1 Conflict of norms

Other conflicts described in the literature pertain to the meso-level. These arise when institutional actors such as the family as a whole, placement agencies or other professional or profit-oriented stakeholders are involved. For example, the live-in carers can find themselves in the ambivalent role of quasi-family members. By placing the live-in carer in this role, families may feel comfortable making requests that go beyond what is stated in the contract. Live-in triads are therefore particularly prone to conflicts between familial norms of comprehensive care and responsibility for relatives on the one hand and contractual agreements between business partners, e.g. regarding free time and specified tasks on the other hand (23). Furthermore, the quality of the relationships within the triad is in danger if no balance between contradicting norms can be found.

#### 3.2.2 Conflict of values

Certain values of placement agencies, like an uncomplicated fungibility of live-in carers from an efficient work force perspective may rather often conflict with values of live-in carers, for instance if they think that good live-in care involves an emotional bond (23).

#### 3.2.3 Conflict of norms *vs*. values

A common conflict of norms vs. values on the meso-level is when family values of unconditional care are at odds with the live-in carer’s right to individual autonomy and privacy. Especially the close relatives tend to lack awareness of the personal rights of the live-in carers (8, 45).

### 3.3 Potential moral conflicts of live-in care on the macro level

#### 3.3.1 Conflict of norms

On the macro-level, political and societal structures as well as legal regulations and principles, e.g. the Aging in place policy of Western welfare states (1), are considered as a level for potential moral conflicts in our framework. At first glance, this level might seem “far away” from the conflicts arising from the everyday communications and negotiations within the triad in the micro-setting of the live-in care arrangement. However, empirical research points to the meaning of the framework conditions of the macro-level and how these come into conflict with familial expectations and contractual agreements regarding the tasks and the working hours of the live-in carer, which may violate national labor law regulations (14, 22). This conflict may arise from the desire for legal employment of a live-in carer, which is not feasible within the existing legal frameworks and in view of the actual care needs. This typical conflict is represented in a much-publicized court trial in Germany (46), in which a live-in carer sued successfully for recognition and remuneration of on-call times, particularly at night. Such a situation may lead to feelings of guilt in family caregivers towards the live-in carer accompanied by fear about potential personal consequences (7).

#### 3.3.2 Conflict of values *vs*. values

Furthermore, state laws manifest values regarding for example the weighing of individual autonomy or privacy which can divert from the live-in carer’s values in this matter. His or her values may rather emphasize the care needs or relation to other family members (7).

#### 3.3.3 Conflict of norms *vs*. values

Another macro-level conflict arises from a disproportion between the extensive care needs that drive live-in arrangements and long-term care legislations, such as in Germany, that does not fully cover these needs. Families then find themselves in a situation where they cannot realize their desire for legal and fair employment with the (financial) resources provided by the system as this would require a two or three-shift live-in care arrangement with more than one live-in carer. However, national labor law still applies. This dilemma is ignored at the macro level, leaving families at the micro level with their feelings of guilt and a conflict of norms and values (8).

In summary, there is a large number of potential moral conflicts that can be enumerated along the indicated axles (level and type of moral conflict). We have only listed some of them and make no claim of the completeness of the table. However, the variations offer an insight into the myriad possible variations of moral conflicts that can arise in the context of live-in care. The framework presented in **Table 1** demonstrates the interconnectivity of the moral conflicts between each societal level and therefore contributes to gaining a multi-perspective understanding of the moral conflicts in the field.

## 4 Discussion and conclusion

In this paper, we have introduced a conceptual framework for the ethical analysis of moral conflicts in migrant live-in care arrangements. We argue that moral conflicts in the context of live-in care can be analyzed as (1) *conflicts between norms*, (2) *conflicts between values*, and (3) *conflicts between norms and values*. All three types of conflicts may occur on the micro-level represented by the intra- and interindividual level of the triad, the meso-level in interactions between agencies and the triad as well as the macro-level with conflicts between legal regulations and policies and the triad. These levels are not independent but interrelated. Especially changes at the macro-level can have a ripple effect, influencing and impacting other levels of the framework.

Due to its rather broad ethical outline based on the fundamental meta-ethical distinction between values and norms, as well as on the differentiation of three societal levels (micro, meso, and macro), the proposed framework is able to accommodate a whole variety of ethical theories and cultural orientations. This is particularly important in a field like migrant live-in care that is located at the intersection of different social spheres and cultural contexts, each connected to specific paradigms of morality. Thus, the intricate web of moral roles and responsibilities that bind the members of a family calls for another ethical perspective and vocabulary than the general rights and obligations regulating interactions between individuals as contractual partners or equal citizens of a political community (14). Furthermore, more individualistic cultural views of morality, for example in the context of modern Western liberalism, prioritize other norms and values than more collectivistic stances that place greater weight on the family or on the community as a whole (44).

The examples provided illustrate the added value of a closer ethical analysis of moral conflicts arising in the live-in care setting. The framework proposed here allows a differentiated characterization and categorization of the concrete evaluative and normative aspects that are at stake in live-care arrangements. It opens moral conflicts arising in live-in care to a differentiated discussion and evaluation according to a whole range of complementary ethical theories and criteria. Especially in view of the triadic care setting, the perspectives of an ethics of care (47, 48) has proven fruitful for more in-depth analyses of the needs, vulnerabilities and asymmetrical relations between the parties involved (12, 23, 49). With regard to the role of the macro-level, human rights-based approaches, for example, regarding labor and migration laws, can highlight important structural perspectives (50, 51). Furthermore, with its different levels, the proposed framework leaves space for cultural differences of moral perspectives and particularly allows to categorize moral conflicts that can arise from the collision of more individualistic and more collectivistic values and norms (44).

Thus, the proposed multi-level framework ultimately demonstrates the complexity of moral conflicts in live-in care that usually involve several parties and their respective culturally embedded moral perspectives, which are located at different (intra- and inter-)individual, institutional, and societal levels. The framework lays the ground for a multi-perspective analysis and provides a heuristic tool to facilitate this in further studies. In doing so it highlights the importance of studying the migrant live-in care arrangement from a triadic perspective rather than an individual perspective. This is because moral conflicts usually are not limited to intra-individual experiences but rather encompass multiple stakeholders within the triad as well as outside of the triad (12, 52, 53). In this context, a central concern is including person in need of care, especially when they have dementia, in the research and ensuring his or her voice is heard. Future research will benefit from expanding the study of moral conflicts even further by extending our perspective beyond the triad to include additional network members. In addition, the study highlights the importance of incorporating the socio-cultural background of all members of the triad in the analysis of moral conflicts.

Although the proposed tool is complex and needs elaboration and discussion, its further development promises several benefits. First, we underline important yet neglected issues of ethics, norms, and values in the care provided to older people by their family members and by live-in carers. Pointing out these issues and bringing them to the attention of social workers, nurses, and other social and health care providers has the potential of assisting these professionals in their efforts to resolve conflicts within the triadic arrangement and to better understand the needs, norms, and values of each of these stakeholders. Moreover, by drawing attention to the different types of conflict that can emerge, we potentially set the ground for future innovative interventions that can be applied at different levels, depending on context and need, ranging from intraindividual to interpersonal, while taking into account institutional sources of stress.

Furthermore, with regard to its practical use, the framework could be applied as a heuristic tool for research and practice to identify the needs and wishes of the individuals in live-in care triads. The framework also can help to prioritize them, for example by highlighting moral questions such as when the care needs of the person with dementia are more important than the work regulations of the live-in carer and when it appears ethically suitable to evaluate the needs of the live-in migrant carer as higher. By supporting the identification and prioritization of problems, the framework can help to define areas of intervention, especially in grave conflicts when the safety of one or more persons involved is at risk.

On the micro-level, the framework could be adapted to be used in interventions and counselling to help actors of the triad to recognize and navigate conflicts of norms and values within themselves and with each other (micro-level). In an adapted version, the framework also has the potential to function as a didactical tool for nurses and social workers to sensitize them for potential conflicts they might observe when working in the context of live-in care arrangements. On the meso- and macro-level, practical application entails advocating for policy changes and legal reforms to align familial expectations and contractual agreements with labor law regulations. Ensuring fair working conditions and protection of live-in carers’ rights can contribute to resolving moral conflicts and promoting ethically acceptable caregiving practices. Additionally, an adapted version of the framework could function as a foundation for ethical recommendations for individuals in the triad but also for agencies and policy makers to better address moral conflicts associated with live-in migrant home care. Finally, it could contribute to public debates, for example, in the media about the moral costs and the acceptability of live-in migrant care as a form of care in industrialized countries.

In sum, the practical application of our considerations involves promoting open communication, ethical reflection, and decision-making within different settings. By adapting and implementing the multi-level conceptual framework in practice, live-in carers, families, organizations, agencies, and policymakers can better understand, address, and resolve moral conflicts within live-in caregiving arrangements. It might facilitate ethical decision-making, policy reforms, and the promotion of fair and respectful caregiving practices.

## Data Availability

The original contributions presented in the study are included in the article/supplementary material, further inquiries can be directed to the corresponding author.
